# Novel 3D UAV Path Planning for IoT Services Based on Interactive Cylindrical Vector Teaching–Learning Optimization Algorithm

**DOI:** 10.3390/s25082407

**Published:** 2025-04-10

**Authors:** Xinghe Jiang, Xuanyu Wu, Zhifeng Zhang, Zhaoxi Hong, Xi Xiao, Yixiong Feng

**Affiliations:** 1 State Key Laboratory of Public Big Data, Guizhou University, Guiyang 550025, China; jiagxh0716@163.com; 2 School of Mechanical Engineering, Guizhou University, Guiyang 550025, China; 3 School of Mechanical Engineering, Zhejiang University, Hangzhou 310027, China; zhzhfeng@zju.edu.cn; 4 State Key Laboratory of Fluid Power and Mechatronic Systems, Zhejiang University, Hangzhou 310027, China; hzhx@zju.edu.cn; 5 Ocean College, Zhejiang University, Hangzhou 310027, China; prana@zju.edu.cn

**Keywords:** UAV, IoT services, 3D path planning, cylinder vector, integrates interactive method, teaching–learning

## Abstract

In the 6G-IoT convergence ecosystem, UAV path planning for static environments is systematically investigated as a resource coordination problem where communication demands and terrain constraints are balanced through intelligent trajectory optimization. The innovation of this paper lies in the proposal of an interactive cylinder vector teaching–learning-based optimization (ICVTLBO) algorithm, where UAV trajectory points are represented in cylindrical coordinates, and targeted interactive strategies are proposed during the teacher and learner phases to address uncertainty challenges, such as terrain elevation fluctuations and communication link instability caused by obstacles in static environments. The ICVTLBO is compared with other classical and novel algorithms on the CEC2022 benchmark function suite, demonstrating its effectiveness and reliability in solving complex optimization problems. Subsequently, real digital elevation model (DEM) maps are utilized to establish nine diverse terrain scenarios for the simulation of 3D UAV path planning challenges, and experimental results show that the ICVTLBO outperforms other methods, providing high-quality paths for UAVs in complex environments.

## 1. Introduction

With the advent of sixth-generation (6G) communication technology, the Internet of Things (IoT) is evolving toward a more intelligent and efficient direction, with significant advancements in seamless connectivity, ultra-low latency, and ultra-high reliability [1]. These developments have accelerated the progress of unmanned aerial vehicle (UAV) technology, particularly with the support of IoT and wireless sensor networks (WSN). In these domains, UAVs have gradually become one of the core technologies supporting emerging application scenarios [2]. They are not only capable of serving dynamic data relays and mobile sensing platforms but also play a crucial role in distributed networks. UAVs provide essential data collection and transmission services in areas such as environmental monitoring and smart cities [3].

However, with the expanding scope of UAV applications, particularly in complex environments, the efficient planning of flight paths has become a pressing issue [4]. In mountainous or other topographically challenging regions, UAVs need to avoid obstacles and effectively exchange data with IoT nodes. At this point, optimizing UAV path planning becomes crucial, as the choice of path directly impacts data collection efficiency and the stability of communication links [5]. For example, in mountainous areas, frequent obstacle avoidance may lead to overly meandering paths, potentially interrupting the communication connection with ground sensors, resulting in data loss or delays. Such situations undoubtedly affect the overall performance of IoT systems, particularly in real-time data transmission and task scheduling scenarios. Recent studies have proposed innovative solutions to improve UAV path planning in such environments. For instance, research on AoI-minimal clustering explores how to minimize the age of information in UAV-assisted networks, thereby enhancing the efficiency of data transmission in IoT systems [6]. Additionally, transmission and trajectory co-design for UAV-assisted wireless powered communication networks (WPCNs) has been investigated to optimize both UAV transmission and movement, leading to more effective data relay and energy utilization [7]. These works underscore the growing demand for integrated path planning algorithms that address both trajectory optimization and communication performance issues in UAV systems.

In this context, the design of efficient UAV path planning algorithms is crucial for IoT applications. Through efficient path planning, unnecessary detours can be minimized, energy consumption during flight can be reduced, and data collection efficiency can be improved, thereby enhancing the overall performance of the IoT system. Especially in complex terrains, good path planning not only ensures efficient obstacle avoidance by UAVs but also provides stable communication between ground sensors and UAVs. Therefore, an efficient path planning algorithm not only helps improve the operational efficiency of UAVs but also has a positive impact on the communication stability of the entire IoT system. Investigations have disclosed that the task of devising optimal path planning for UAVs is subsumed within the class of NP problems that are exceedingly challenging, a complexity that is observed to escalate exponentially in direct correlation with the augmentation of the problem’s dimensions [8,9,10].

Recently, a significant surge in research has led to the development of numerous path planning algorithms specifically designed for UAV operations. Among these, metaheuristic algorithms based on swarm intelligence have gained prominence, playing a vital role in advancing UAV path planning methodologies. For instance, Cunjie Li et al. [11] proposed an improved particle swarm optimization algorithm combined with a genetic algorithm, which strengthens the global search capability and enhances local optimization, enabling the UAV to find the shortest path and avoid all threats in both simple and complex terrain environments. Cao et al. [12] proposed a UAV route planning method based on genetic algorithms. Experimental results demonstrate that the improved genetic algorithm, based on artificial intelligence, is both efficient and feasible for autonomous UAV route planning. Van Truong Hoang et al. [13] proposed an optimization algorithm called multi-subject-TLBO, which enhances the original method through mutation, elite selection, and multi-agent training to improve solution quality and accelerate convergence. This approach effectively generates optimal, collision-free, and feasible flight paths for UAVs in complex operational environments. Although existing UAV path planning algorithms can meet basic flight requirements to some extent, there is an urgent need to develop more advanced planning algorithms as task complexity increases and environmental uncertainty intensifies.

The teaching–learning-based optimization (TLBO) algorithm, as initially proffered by Rao et al. in 2011 [14], has been widely recognized as an efficient population-based method that mimics knowledge transfer mechanisms in classroom environments. While its parameter-free structure and rapid convergence characteristics have enabled successful applications in power dispatch and production systems, three fundamental limitations persist in complex optimization scenarios: premature convergence due to population diversity loss, inadequate environmental adaptation mechanisms, and insufficient collaborative search strategies [15]. Recent advancements in TLBO variants have systematically addressed these challenges through two primary improvement pathways. First, TLBO algorithm operators are modified to enhance search flexibility, as demonstrated by Hong et al. [16] through reliability-based teaching factors that enable phased convergence control. Wang et al. [17] proposed an improved DSTLBO algorithm that uses dynamic opposition populations, a sorting mechanism for learning styles, and random information exchange to enhance exploration and avoid local optima, significantly improving the efficiency of photovoltaic parameter extraction. Second, hybrid strategies have been developed to combine TLBO with complementary optimization philosophies, for example, Santosh Kumari Meena et al. [18] presented a hybrid TLBO-PSO method to minimize PMU installation while ensuring full system observability; Kundu and Garg [19] presented the integration of Levy-flight mutation operators from SMA algorithms. Thanh Long Duong et al. [20] proposed a hybrid approach DE-TLBO to simultaneously optimize reactive power planning and determine the optimal size of the thyristor-controlled series compensator (TCSC), thereby effectively minimizing power losses in the ACTEP problem. In summary, integrating various improvement strategies into the TLBO algorithm has significantly enhanced its optimization capabilities. As a result, both the original and improved versions of the algorithm have demonstrated widespread applicability across diverse engineering and operational challenges, encompassing though not limited to improvements in economic load dispatch [21], smart city communication and energy management [22], optimal distribution of reactive power within electric grids [23], production systems [24], logistics and supply chain management issues [25], nonlinear equation problems [26], shop scheduling problems [27], high-dimensional expensive problems [28], and optimal control systems [29].

However, despite the advancements made in these enhanced algorithms, issues such as slow convergence and insufficient solution accuracy persist, especially in the rapidly evolving context of the IoT and increasingly complex environments. These limitations hinder the practical application and broader adoption of such algorithms. Furthermore, the application of TLBO to UAV path planning remains relatively underexplored, contributing to a significant technological bottleneck in this field. To address this gap, this study introduces an innovative solution—an innovative interactive cylindrical vector teaching–learning-based optimization (ICVTLBO) algorithm. Designed to tackle the challenges of 3D UAV path planning in complex environments, ICVTLBO aims to improve both the efficiency and accuracy of path planning. The main contributions of this paper are as follows:

1. The ICVTLBO algorithm is proposed in this study, which achieves high-precision 3D path planning in complex terrains while maintaining computational efficiency. Its low complexity architecture and adaptive search mechanism lay the foundation for future integration with communication-aware optimization, which is essential for UAV functioning as persistent mobile base stations in IoT networks.
2. Considering the dynamic nature of drone path planning, waypoints are represented using polar coordinates. The interaction strategies between the teacher and learner phases enhance group diversity and global search capability, accelerating convergence and improving solution accuracy, ensuring the ICVTLBO algorithm effectively plans smooth optimal paths in complex environments. When drones are deployed as IoT data relays, this method is crucial for maintaining network coverage in remote monitoring scenarios.
3. The effectiveness and robustness of the ICVTLBO algorithm are validated through experimental comparisons with several leading algorithms on the 20-dimensional problems from the CEC2022 benchmark function suite. Further validation across nine different terrain scenarios demonstrates its adaptability and superiority, showcasing its ability to plan high-quality paths for UAVs in complex 3D environments. The algorithm’s high performance ensures reliable UAV operation in diverse terrains, particularly in mountainous wireless sensor network clusters, without the need for environment-specific adjustments.

The subsequent parts of this paper are composed as follows: Section 2 focuses on the problem of UAV path planning. Section 3 introduces the teaching–learning-based optimization algorithm and proposes the improved ICVTLBO algorithm. Section 4 validates the ICVTLBO algorithm through numerical verification experiments. Section 5 presents an experimental analysis of UAV path planning. Finally, Section 6 provides a summary of this paper.

## 2. Problem Modeling and Mathematical Description

This section primarily elaborates on the mathematical model of UAV path planning, providing a detailed theoretical foundation for subsequent research through rigorous mathematical language and logical reasoning.

### 2.1. Route Representation

In the context of mission space, if O and D are designated as the initial position and endpoint, respectively, the planned path of the UAV is expressed through a tandem of M waypoints inserted between O and D. The UAV’s path is then expressed as a series of M waypoints. This construction can be mathematically encapsulated as:

(1) $$\mathrm{Route}=OPcup_{i=1}^{M}{\mathrm{WP}}_{i}\cup DP$$

where OP denotes the starting point (O), DP denotes the goal point (D), ${\mathrm{WP}}_{i}$ denotes the i th waypoint, and M signifies the aggregate number of waypoints.

### 2.2. Evaluation Function

The evaluation function serves as a metric for quantifying the “quality” or “merit” of specific aspects. When considering the flight characteristics of drones and safety factors during flight, the evaluation function for the flight path P can be constructed by assessing four cost elements: path length cost, flight altitude cost, threat cost, and the maneuver constraint of the UAV. These cost elements collectively constitute the evaluation function, denoted as Y, whose specific expression is as follows:

(2) $$Y=\sum_{n=1}^{4}K_{n}\kappa_{n}$$

where the evaluation function Y is a composite measure that considers various cost factors associated with the flight path P. Specifically, $K_{n}\;(n=1,\ldots ,4)$ represent the costs attributed to the path length, the flight altitude, the threats, and the maneuver constraints of the UAV, respectively. The coefficients $\kappa_{1}$ through $\kappa_{4}$ are the respective weightings for each of these cost components $K_{1}$ through $K_{4}$; their value size is usually based on experience, requirement $\kappa_{1}+\kappa_{2}+\kappa_{3}+\kappa_{4}=1,\kappa_{n}\neq 0$.

#### 2.2.1. Path Length Cost

The desired flight route for the UAV is anticipated to be as concise as feasible to conserve fuel. This is quantified by the aggregation of multiple segment lengths. The Euclidean distance between any two nodes is adopted as the length of an individual segment. Consequently, the cost associated with the path length for a given path can be computed as follows:

(3) $$K_{1}(P_{i})=\sum_{i=0}^{N}\sqrt{\left[{\left(x_{i+1}-x_{i}\right)}^{2}+{\left(y_{i+1}-y_{i}\right)}^{2}+{\left(z_{i+1}-z_{i}\right)}^{2}\right]}$$

where the i flight path $P_{i}$ coordinate is $\left(x_{i},y_{i},z_{i}\right)$.

#### 2.2.2. Flight Altitude Cost

Maintaining the aerial security of the UAV necessitates navigating at an optimal altitude. Operating at excessive heights can increase the likelihood of radar detection, highlighting the importance of exploiting terrain features to minimize such risks. Conversely, flying too low poses the hazard of collision with terrain elevations or other obstructions. Consequently, the flight height cost of the UAV can be expressed as follows:

(4) $$K_{2}(P_{i})=\left\{\begin{array}{cc} \left|h_{i}-\frac{\left(h_{\max}+h_{\min}\right)}{2}\right|, & h_{i}\geq h_{\min}\\ A_{T}, & h_{i}<h_{\min} \end{array}\right.$$

where $h_{i}$ represents the height of the i th flight path $P_{i}$, $h_{\min}$ denotes the minimum altitude at which the UAV can fly, $h_{\max}$ denotes the maximum altitude at which the UAV can fly, and $A_{T}$ is the penalty value when the flight height is lower than the $h_{\min}$.

#### 2.2.3. Threat Cost

In UAV path planning, threat cost refers to the risk associated with the presence of potential threats along a drone’s flight path. These threats could be obstacles, hostile entities, or other dangerous factors that might compromise the UAV’s safety during its mission. The threat cost function aims to quantify and minimize the risk by considering the proximity of the UAV to these threats. The threat cost associated with route $P_{i}$ is articulated as:

(5) $$K_{3}(P_{i})=\left\{\begin{array}{l} (r_{u}+\mathbb{Q})-d_{u},0<d_{u}-r_{u}\leq \mathbb{Q}\\ 0,d_{u}-r_{u}>\mathbb{Q}\\ A_{T},d_{u}-r_{u}\leq 0 \end{array}\right.$$

where u represents the quantity of threats present, $r_{u}$ signifies the core locus of the u th threat source, $d_{u}$ is the distance from the drone to the center of the u th threat source, and ℚ indicates a safe distance.

#### 2.2.4. The Maneuver Constraint of UAVs

The aeronautical maneuverability of the UAV is primarily dictated by the turning angle and the slope angle—critical metrics that ascertain the feasibility of a flight path. The turning angle is defined by the angulation between the horizontal Oxy projections of two successive line segments, which is calculated as follows:

(6) $$\psi \geq \arccos(\frac{{\left(x_{i}-x_{i-1},y_{i}-y_{i-1}\right)}^{T}(x_{i+1}-x_{i},y_{i+1}-y_{i})}{\left|(x_{i}-x_{i-1},y_{i}-y_{i-1})|\cdot |(x_{i+1}-x_{i},y_{i+1}-y_{i})\right|})$$

where $\left(x_{i}-x_{i-1},y_{i}-y_{i-1}\right)$ represents the horizontal projection of the UAV’s flight path.

Like the slope angle, which plays a crucial role in influencing drone flight stability, the maximum pitch angle refers to the maximum pitch angle the drone’s body can reach when transitioning from the current waypoint to the next in the vertical direction. The flight process of a drone involves complex changes in motion states, with climbing and diving being key actions. During the climb phase, the drone must raise its nose to generate upward lift, thereby increasing its altitude. In the dive phase, the nose is lowered to utilize gravity for rapid descent. To ensure that the drone does not experience collisions or catastrophic failures, such as crashes or falls during climbing, diving, or other flight processes, constraints must be imposed on the pitch angle. The actual pitch angle should always be less than or equal to the pre-set maximum pitch angle. Therefore, the mathematical expression is as follows:

(7) $$\Omega \geq \arctan(\frac{\left|z_{i+1}-z_{i}\right|}{\left|(x_{i+1}-x_{i},y_{i+1}-y_{i})\right|})$$

The physical constraint of the UAV can be expressed as follows:

(8) $$K_{4}=\sum_{i=1}^{N-1}d\psi_{i}+\sum_{i=0}^{N}d\Omega_{i}$$

where $d\psi_{i}$ is the loss caused by the angle of the flight path $P_{i}$, and $d\Omega_{i}$ is the loss caused by the slope angle of the flight path $P_{i}$. $\psi_{\max}$ is the maximum turning angle, and $\Omega_{\max}$ is the maximum pitch angle; if $\psi_{i}>\psi_{\max}$, then, $d\psi_{i}=0$; if the $\psi_{i}\leq \psi_{\max}$, then $d\psi_{i}=\psi_{i}$; if $\left|\Omega_{i+1}-\Omega_{i}\right|\leq \Omega_{\max}$, then $d\Omega_{i}=|\Omega_{i+1}-\Omega_{i}|$, otherwise $d\Omega_{i}=0$.

## 3. Teaching–Learning-Based Optimization (TLBO) Algorithm

In this section, the fundamental TLBO algorithm is initially presented. Central to this algorithm are two primary roles: teachers and students. Teachers are tasked with instruction, employing a variety of methods to impart knowledge and skills, as well as guiding the growth and development of students. As educators, they are required not only to have a solid foundation in their subject matter but also to possess exceptional teaching techniques and communication abilities for effective instruction. Students, on the other hand, are the recipients of education; they acquire new knowledge and enhance their overall capabilities by learning from teachers, outstanding peers, and through interactive learning experiences.

Within the field of UAV path planning, maneuverability is crucial for flight efficiency and safety. The traditional Cartesian coordinate system fails to adequately describe the dynamic characteristics of drones and obstacle avoidance. To address this, a cylindrical coordinate system approach is proposed in this paper, utilizing its features to express drone motion and simplify spatial constraints. Building upon this, an enhanced version of the TLBO algorithm is presented, integrating a variety of targeted interactive strategies. These strategies are designed to amplify the speed and improve the quality of solutions, ultimately facilitating the attainment of superior flight paths for UAVs.

### 3.1. Teacher Phase 

In the teacher phase, an emulation of the instructional processes is conducted to identify the solution with the most optimal objective function value within the cohort, a process that can be modeled using Equation (10):

(9) $$Q_{j}^{new}=S_{j}^{i}+\mathrm{rand}\times \left(S_{j}^{best}-T_{F}\times S_{j}^{Mean}\right)$$

where $Q_{j}^{new}$ and $S_{j}^{i}$ are utilized to denote the positioning of an entity after and before learning, respectively. The term rand refers to a random number encompassed by the interval [0, 1]. $S_{j}^{best}$ represents the location of the teacher, which is ascribed to the most outstanding student entity within the classroom cohort. $S_{j}^{Mean}$ signifies the mean level of the search agents present in the entirety of the population. $T_{F},$ identified as a teaching factor, plays a pivotal role in modulating the value of $S_{j}^{Mean}$. The potential value of $T_{F}$ can be either 1 or 2, determined stochastically based on the probability distribution stipulated $T_{F}=round(1+rand\left(0,1\right)\left\{2-1\right\}$.

### 3.2. Learner Phase

In addition to gleaning knowledge from the teacher, learners can also enhance their understanding by learning from each other. In the context of collaborative learning, a learner can internalize knowledge from a randomly chosen peer who occupies a superior rank. The strategic approach of the learner may be formulated as follows:

(10) $$S_{j}^{new}\;=\left\{\begin{array}{l} S_{j}^{i\;}+r\times \left(S_{j}^{\mathrm{rand}\;}-S_{j}^{i}\right),f\left(S_{j}^{i}\right)<f\left(S_{j}^{\mathrm{rand}}\right)\\ S_{j}^{i\;}+r\times \left(S_{j}^{i}-S_{j}^{\mathrm{rand}}\right),\;\mathrm{otherwise} \end{array}\right.$$

where $S_{j}^{\mathrm{new}\;}$ denotes the position of the learner after learning, $S_{j}^{\mathrm{rand}\;}$ is the position of a student randomly selected from the class, and r is a random number between [0,1].

### 3.3. Improved TLBO Algorithm for Path Planning

In this section, a cylindrical coordinate system model is proposed first, and then, we endeavor to enhance the search efficiency of the TLBO algorithm when addressing optimization problems. To achieve this, we have meticulously refined and augmented the algorithm’s core components, namely, the teaching and learning phases—while preserving their inherent strengths such as simplicity and ease of implementation. Specifically, we have devised targeted interactive strategies, tailored to cater to the distinct requirements of the teaching and learning processes. These innovations have significantly bolstered the population’s exploration and exploitation capabilities, which are critical for avoiding local optima and pinpointing the global optimum. The dual-phase, interactive enhancements ensure that the algorithm navigates the search space more effectively, thereby increasing the likelihood of converging on solutions of superior quality.

#### 3.3.1. UAV Path Planning Method Based on Cylindrical Vector

In the study of UAV path planning, maneuverability is of paramount importance as it affects the efficiency and safety of UAV flight in complex environments. The traditional Cartesian coordinate system has been identified to have limitations when describing the dynamic characteristics of UAVs and their obstacle avoidance capabilities. Consequently, a novel approach based on a cylindrical coordinate system is presented in this paper, which encodes each feasible path from the starting point to the endpoint as a series of vectors. Within this framework, an individual vector meticulously defines the UAV’s flight maneuver as it transitions from one waypoint to the subsequent one. In the UAV path planning method premised on cylindrical vectors, each vector in the set comprises three components: ρ∈(0,path_length),θ∈(−π/2,π/2), and ϕ∈(−π,π). Here, ρ signifies the magnitude, θ denotes the flight elevation angle of the UAV, and ϕ corresponds to the UAV’s azimuthal orientation. Consequently, any viable path $\Omega_{i}$ with n navigational nodes can be articulated by a compilation of 3D cylindrical vectors, as illustrated below:

(11) $$\Omega_{i}=(\rho_{i1},\theta_{i1},\phi_{i1},\rho_{i2},\theta_{i2},\phi_{i2},\cdots ,\rho_{in},\theta_{in},\phi_{in})$$

The fundamental principle underlying the cylindrical vector-based path planning approach involves leveraging the interplay between the magnitude, elevation, and azimuth angles experienced during UAV flight and the corresponding velocity, turning angle, and climb angle of the UAV. This method facilitates the enhancement of both safety and efficiency in the planned path. By enabling metaheuristic algorithms to operate within the configuration space rather than Cartesian space, the cylindrical vector-based technique effectively curtails the search domain, thereby augmenting the algorithm’s capacity to discern superior flight paths for the UAV.

The projection P of the waypoint M on the Oxy plane needs to be determined. Subsequently, the cylindrical coordinates can be transformed into Cartesian coordinates through the following equations.

(12) $$\left\{\begin{array}{l} x_{j}=x_{j-1}+r_{j}\cos\theta_{ij}\\ y_{j}=y_{j-1}+r_{j}\sin\theta_{ij}\\ z_{j}=z_{j-1}+\Delta z_{j} \end{array}\right.$$

where $\left(x_{j-1},y_{j-1},z_{j-1}\right)$ and $\left(x_{j},y_{j},z_{j}\right)$ are the waypoint $p_{j-1}$ and $p_{j}$ in the Cartesian coordinate system, and (r,θ,z) is the cylinder vector.

#### 3.3.2. Interactive Strategy at the Teacher Phase

In actual teaching, teachers fulfill a pivotal role. Their responsibilities extend beyond a profound command of subject matter to include exceptional pedagogical skills and communicative abilities, enabling them to efficiently impart knowledge to students. To cater to the diverse learning needs of their pupils, teachers must employ a versatile repertoire of instructional methods and strategies. Furthermore, timely assessment and feedback on student progress are essential, as they inform adjustments to the content and approach of teaching, thereby facilitating continuous improvement in educational practice.

Interaction between teachers and learners is a critical component of the educational process, fostering not only the transfer of knowledge but also enhancing student engagement and motivation. Nevertheless, individual differences among learners, such as variations in cognitive styles, learning habits, and prior knowledge, can impede the complete absorption and comprehension of the knowledge by learners. The dynamic process is defined as follows:

(13) $$Q_{j}^{\mathrm{updated}}=S_{j}^{i}+ℑ\times \varsigma \times \left(S_{j}^{\mathrm{best}\;}-T_{F}\times S_{j}^{Mean}\right)$$

(14) $$ℑ=\left[e^{\frac{-iter}{MaxIt}}+(\cos(\pi \times \frac{iter}{MaxIt})+1)\right]\times \partial$$

where $Q_{j}^{\mathrm{updated}}$ is the position of the individual after learning, and ς is random numbers between (0,1). ∂ is a constant of 0.67, iter denotes the current number of iterations, and MaxIt denotes the maximum number of iterations.

**Figure 1:**
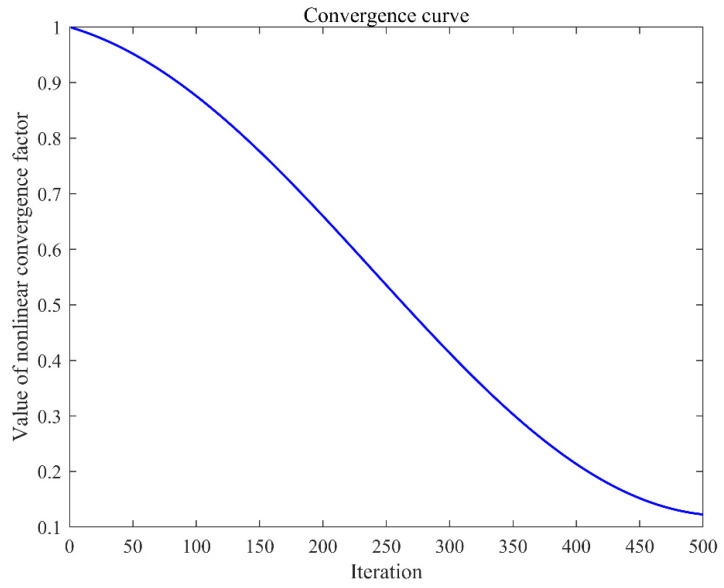
Nonlinear convergence factor.

Figure 1 illustrates a nonlinear decline in the value of ℑ as the number of iterations increases. This trend mirrors a fundamental aspect of the teaching process: initially, students possess a limited knowledge base, offering substantial room for improvement, which results in notably effective teaching interactions. As teaching progresses, and students develop a solid foundation, the potential for significant advancement through interaction diminishes. In response to this observation, the refined algorithm decelerates the performance value’s decay at the onset of iterations, enabling more extensive global exploration. Later in the iterative process, the algorithm accelerates convergence, enhancing the efficiency of the global search. This modified teaching phase effectively boosts the overall performance of the algorithm.

#### 3.3.3. Enhanced Interactive Learner Phase

On the foundation of previous research into the learner phase, this paper further contemplates the significant impact of student interactions during the educational process on learning outcomes. In practical education, in-depth exchanges and communication with students who excel academically play an undeniable role in enhancing learning effectiveness. There is a positive correlation between the frequency of interaction and learning outcomes, which stems from the fact that by interacting with high-performing peers, students can acquire a richer reservoir of knowledge and valuable experience, and receive positive encouragement and guidance, thereby effectively boosting their motivation to learn and improving their academic performance. Moreover, spatial proximity is another crucial factor affecting learning efficacy. Maintaining proximity to top-performing students allows for more convenient seeking of help and advice, consequently gaining access to more learning resources and support. At the same time, close interaction with outstanding students helps ignite enthusiasm for learning and uncovering one’s potential. The above processes can be defined mathematically as follows:

(15) $$Le_{j}^{\mathrm{updated}\;}=\left\{\begin{array}{l} S_{j}^{best}+\left[\hbar \times \sin\left(\frac{iter\times rand}{MaxIt}\right)\times \left|rand\times S_{j}^{i}-S_{j}^{best}\right|\right]\times Levy\left(Dim\right),\;if\;\mathrm{rand}<0.5\;,\\ S_{j}^{best}+\left[\hbar \times \cos\left(\frac{iter\times rand}{MaxIt}\right)\times \left|rand\times S_{j}^{i}-S_{j}^{best}\right|\right]\times Levy\left(Dim\right),\;\mathrm{otherwise} \end{array}\right.$$

The learning rate adjustment, ℏ, is influenced by both the iteration number and random variables, ensuring that the algorithm adapts dynamically across iterations. This term can be described as follows:

(16) $$\hbar =\;\;\left(\mathrm{mod}\left(3.468\times rand\times \left(1-rand\times \cos\left(\arccos(rand\times 10^{4})\right)\right),1\right)\right)$$

where rand represents a random number between 0 and 1; the value of ℏ is [0, 1], affecting the exploration–exploitation balance of the algorithm; and Levy(Dim) [30] is used to denote the impact of the distance between students on the effectiveness of their interactions, as specified by the following equation:

(17) $$Levy(Dim)=0.01\times (rand\times \left(\frac{\Gamma (1+\iota )\times \sin(\pi \iota /2)}{\Gamma (\frac{1+\iota }{2})\times \iota \times (2^{\frac{\iota -1}{2}})}\right))/(|v^{\left(1/\alpha \right)}|)$$

where the ι=1.5 is a constant representing the scale of the Levy flight, and v and α are randomly selected numbers in the range of [0, 1]. This random walk mechanism, described by the Levy distribution, is crucial for balancing the global exploration and local exploitation aspects of the algorithm.

**Figure 2:**
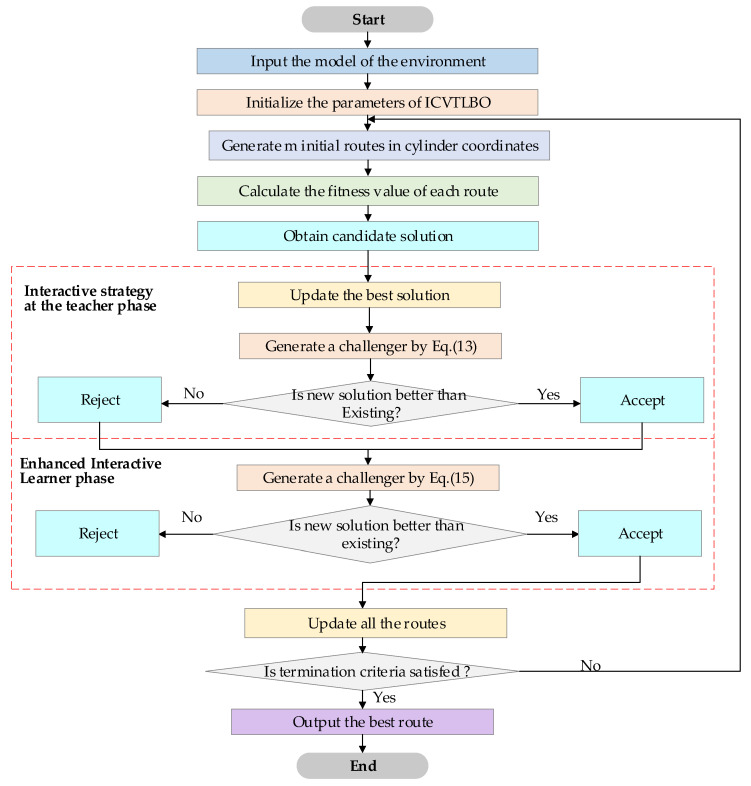
Flowchart of ICVTLBO.

In summary, the flow chart of ICVTLBO is shown in Figure 2. The specific processes are as follows:

Step 1: The environment model, including the terrain, obstacles, and any other environmental constraints, is input into the system. This model will be used throughout the optimization process to simulate and evaluate the paths.

Step 2: Key parameters of the ICVTLBO are initialized, including population size, maximum iterations, and cylindrical coordinate system parameters.

Step 3: Initial UAV routes are randomly generated in cylindrical coordinates, leveraging rotational maneuver characteristics of UAVs. Each path is represented as a vector with angular and radial components.

Step 4: The fitness value of each path is calculated based on criteria, including path length, collision risk with static obstacles, and terrain clearance safety margin.

Step 5: The population is ranked by fitness values, and the top-performing path is designated as the preliminary candidate solution.

Step 6: Step 6a: A teacher-guided challenger path is generated using Equation (13), which integrates terrain elevation constraints into the cylindrical vector update mechanism. Step 6b: The challenger’s fitness is compared with the current candidate solution. If superior, the challenger replaces the current solution; otherwise, it is rejected.

Step 7: The global best path is iteratively refined based on the teacher phase outcomes, ensuring alignment with static environmental features.

Step 8: Step 8a: A neighborhood-based challenger path is generated via Equation (15), incorporating dynamic vector adjustments between adjacent UAV trajectories to avoid local optima. Step 8b: The challenger is accepted only if it demonstrates improved fitness, emphasizing obstacle avoidance and path smoothness in static environments.

Step 9: All UAV paths are synchronously updated based on the interactive teacher–learner phases, preserving diversity through cylindrical vector rotations and radial scaling.

Step 10: The process repeats until predefined termination criteria.

Step 11: The algorithm outputs the globally optimal collision-free 3D path in Cartesian coordinates, optimized for static terrain constraints and IoT service requirements.

## 4. Numerical Simulation Results Analysis

The performance of the ICVTLBO algorithm introduced in this investigation is evaluated through a comprehensive array of comparative experiments aimed at confirming its potential superiority over both established and innovative meta-heuristic optimization methods. In this vein, the study utilizes the recently published CEC2022 benchmark suite [31,32]—a preeminent and challenging collection of test functions within the evolutionary computation domain—as the standard for assessment.

### 4.1. Competing Algorithms and Parameters Setting

In the performance evaluation of this study, the difficult 20-dimensional problem in the CEC2022 benchmark function is selected as the evaluation environment to perform an in-depth comparative analysis of the proposed ICVTLBO algorithm. The lineup of comparison algorithms covers particle swarm optimization [33] (PSO), the artificial bee colony algorithm [34] (ABC), the improved dwarf mongoose optimization algorithm [35] (IDMO), the coati optimization algorithm [36] (COA), the TLBO algorithm [14], and the teaching–learning-studying-based optimization algorithm [37] (TLSBO). Among them, PSO and ABC are classical algorithms in the field of metaheuristics, while IDMO and COA represent the latest research progress, and TLSBO is an improved version based on TLBO. To ensure the fairness of the comparison, the maximum number of iterations of all the algorithms in the experiment is uniformly set to 500, and the population size is 30 individuals. To improve the credibility of the results, each experiment was repeated independently for 30 rounds to reduce the influence of random variation on the results and to provide sufficient data for subsequent statistical analysis. A series of simulation experiments were conducted using MATLAB 2021b on a computer equipped with an AMD Ryzen 53550H CPU @2.10 GHz and 16G RAM, running on a 64-bit Windows 10 operating system.

This study extends beyond the mere average algorithmic performance, incorporating an interrogation of each algorithm’s concordance via the calculation of standard deviations. In addition, a nonparametric statistical tool—the Friedman rank sum test [38,39]—further confirms the veracity of the performance differences between the algorithms. The test, which requires no initial assumptions about data distribution, is particularly well suited for dealing with datasets that deviate from normality, making it a powerful tool for measuring the efficacy of optimization algorithms. The parameters setting of each algorithm involved in the comparison are set according to the values given in the reference.

### 4.2. Compare Using CEC 2022 Benchmark Functions

Table 1 presents the performance metrics for each algorithm on the CEC2022 benchmark suite. The ‘Ave’ row shows the mean values from 30 independent runs, while ‘Std’ represents the standard deviation. The last three rows include ‘W|T|L’, indicating the count of functions where an algorithm achieved superior (win), statistically equivalent (tie), or inferior (loss) performance; ‘Mean’, showing the average values from the Friedman rank-sum test; and ‘Ranking’, which lists the overall rankings of the algorithms. Bold highlights the optimal values.

In the CEC2022 20-dimensional benchmark tests, the ICVTLBO algorithm exhibits strong performance. Although it lags competitors in four functions (F2, F6, F10, and F12), it outperforms them in the remaining eight functions, highlighting its effectiveness and versatility in solving complex problems. Additionally, ICVTLBO yields the optimal Std in six functions, with Std values in the other functions being slightly inferior to those of other contenders. However, overall, the cumulative number of optimal Std values achieved by ICVTLBO far surpasses those of the compared algorithms. The (W|T|L) statistics indicate that ICVTLBO attains the best results in eight functions, without any worst performances. The Friedman ranking value for ICVTLBO in 20-dimensional is 1.5417, positioning it at the top and significantly ahead of all other compared algorithms. It is noteworthy that TLBO and its variants also produce remarkable results, underscoring the research significance of TLBO. In conclusion, despite fluctuations in stability observed in certain test sets, ICVTLBO exhibits a pronounced advantage in global optimization capabilities and overall performance. This affirms its potential in addressing complex optimization issues and lays a solid foundation for future algorithm enhancements and applications.

**Figure 3:**
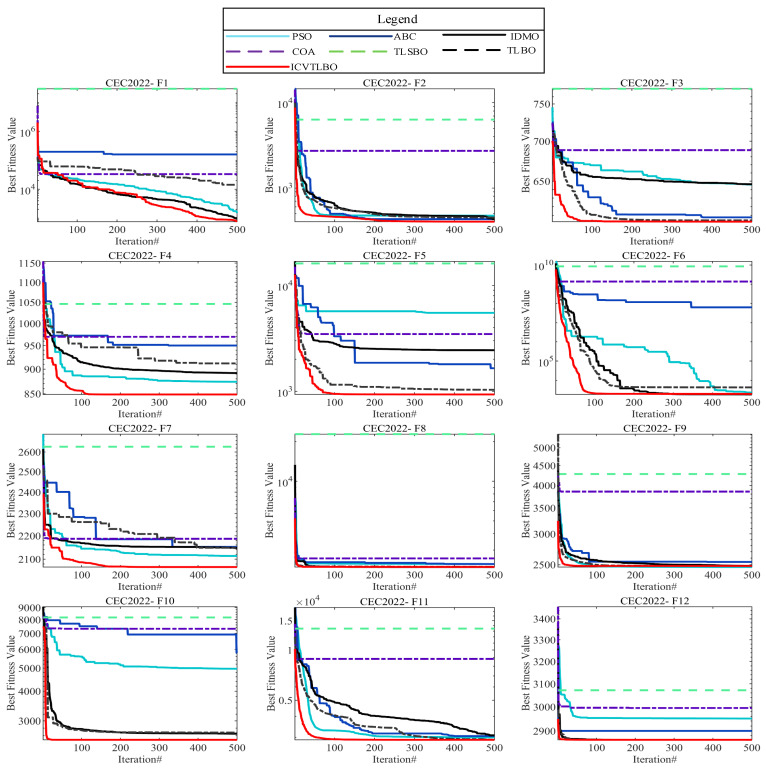
Comparison of the convergence speed of each algorithm in CEC2022-20 dimensionality.

As depicted in Figure 3, the convergence curves of the CEC2022 algorithm performance assessments vividly illustrate the optimization efficacy of various algorithms. Through an in-depth analysis of the convergence behaviors within the CEC2022 benchmark suite, a detailed comparison of their optimization capabilities has been conducted. The findings reveal that on five test functions—F1, F3, F4, F5, and F7—other algorithms are prone to local optima in contrast to ICVTLBO. Within the 12 tested functions shown in Figure 3, the ABC, COA, and TLSBO algorithms tend to be locally optimal in all function tests. These limitations suggest that these algorithms face significant challenges in the global search process and are prone to fall into local optima. In stark contrast, the ICVTLBO proposed herein demonstrates remarkable performance enhancements across 12 test function convergence curves. ICVTLBO achieves substantial progress in both convergence speed and the precision of the final solutions. These outcomes not only underscore ICVTLBO’s exceptional global exploration abilities during the optimization process but also validate its effectiveness in enhancing both convergence speed and accuracy. This serves as a testament to the success of the strategies introduced in this study in addressing the shortcomings of the traditional TLBO algorithm in these areas.

Through such refinements, ICVTLBO significantly augments its performance in handling complex optimization problems while preserving algorithmic simplicity, offering a robust optimization tool for research endeavors within the relevant fields.

## 5. UAV Path Planning Experiments

To thoroughly assess the efficacy of the proposed algorithm, a series of simulations are executed upon authentic digital elevation model (DEM) maps that are sourced from LiDAR sensor-derived terrain data [40]. These simulations entail the development of nine distinct UAV path planning scenarios, each presenting a unique level of terrain complexity, thereby allowing for a comprehensive evaluation of the algorithm’s performance. Within this experimental context, a series of comparative analyses are conducted, positioning ICVTLBO against well-established optimization methods, including PSO, ABC, TLBO, TLSBO, and COA. Central to these analyses is the optimization of a carefully devised cost function, which serves as the basis for quantifying and comparing the optimization proficiency of each algorithm in addressing path planning challenges. Throughout the optimization endeavor, all contending algorithms utilize a cylindrical vector planning methodology for pathway search and refinement. In this process, prospective flight paths are initially encoded into a collection of vectors—defined by their amplitude, elevation angle, and azimuth—utilizing a metaheuristic encoding technique. This is followed by an extensive search for the configuration space to explore these vectors. Upon completion of the search, the resultant vectors are then converted into a set of Cartesian coordinates, permitting a heightened precision in the delineation of three-dimensional pathways. Concluding the procedure, the cost function is applied to each candidate pathway, culminating in the identification of the path exhibiting the least cost as the most optimal solution.

### 5.1. Experimental Settings

A fixed parameter set is utilized across the experiments to ensure consistency. The population size is designated as N = 100 to facilitate iterative optimization, and the upper limit for iterations is set at MaxIter = 200, which strikes a balance between exhaustive search and computational efficiency. Moreover, the number of pathway nodes is established at *n* = 10, forming the scaffold for the drone’s flight path. The starting coordinates are defined as (200,100,150), with the endpoint specified at (800,800,200), reflecting the unipoint-to-unipoint nature of the flight mission. Each simulation scenario is run independently 30 times, with outcomes systematically documented in tabular form for subsequent analytical purposes. Furthermore, nine distinct terrain scenarios have been meticulously crafted, each of which includes a series of obstacles, all represented as red cylinders. The three-dimensional coordinates and radii of these obstacles are meticulously listed in Table 2, providing crucial information for understanding UAV flight path planning in diverse landscapes.

### 5.2. Comparison and Analysis of Results of Different Methods

The performance data of the six optimization methods in 30 rounds of independent testing are summarized in Table 3, with the superior outcomes being emphasized in boldface type. The data reveal that ICVTLBO consistently exhibits superior accuracy across all tested scenarios, surpassing the performance of other comparative algorithms.

Within scenarios 1 to 4, characterized by fewer obstacles and threats, the average fitness values among the various methods do not deviate significantly. For instance, in scenario 2, ICVTLBO achieves an average fitness value of 4627.4116, whereas the corresponding values for TLBO, TLSBO, PSO, and ABC are 4645.3865, 4655.3958, and 4646.9727, respectively. Notably, ICVTLBO demonstrates remarkable stability as its standard deviation in fitness value is merely 0.8281, a figure distinctly lower than the standard deviations of the other compared methods, thereby affirming its significant advantage in terms of stability.

Enhanced scrutiny of the data encapsulated in Table 3 indicates a decline in solution precision for the other algorithms, aside from ICVTLBO, as the complexity of scenarios escalates from 5 to 9, encompassing a greater number of obstacles. Within these intricate environments, although the COA algorithm maintains a certain level of solution quality, it fails to parallel ICVTLBO’s performance. Specifically, ICVTLBO records average fitness values of 5160.3514, 4888.1636, 5413.4242, 5131.2233, and 5209.7683 across scenarios 5 to 9, respectively. These figures unequivocally demonstrate ICVTLBO’s efficacy and stability in addressing complex path planning problems, even amidst a general decline in search capabilities.

Upon examining Table 3, the superior performance of the ICVTLBO algorithm becomes evident. Specifically, in the third-to-last row (W|T|L), the algorithm consistently achieves the best performance across all nine scenarios. In contrast, the PSO algorithm demonstrates inferior performance across these scenarios, while the other algorithms tested exhibit moderate performance levels. Furthermore, as indicated by the Friedman rank sum test results presented in the last two rows of Table 3, the ICVTLBO algorithm ranks first with a significantly lower mean rank compared to the other algorithms, affirming its leading position within the comparative group.

In summary, through a comparative analysis against other advanced algorithms, ICVTLBO proves to have a significant edge not only in the quality of solutions but also in the stability and reliability of the algorithm when tackling diverse path planning challenges.

**Figure 4:**
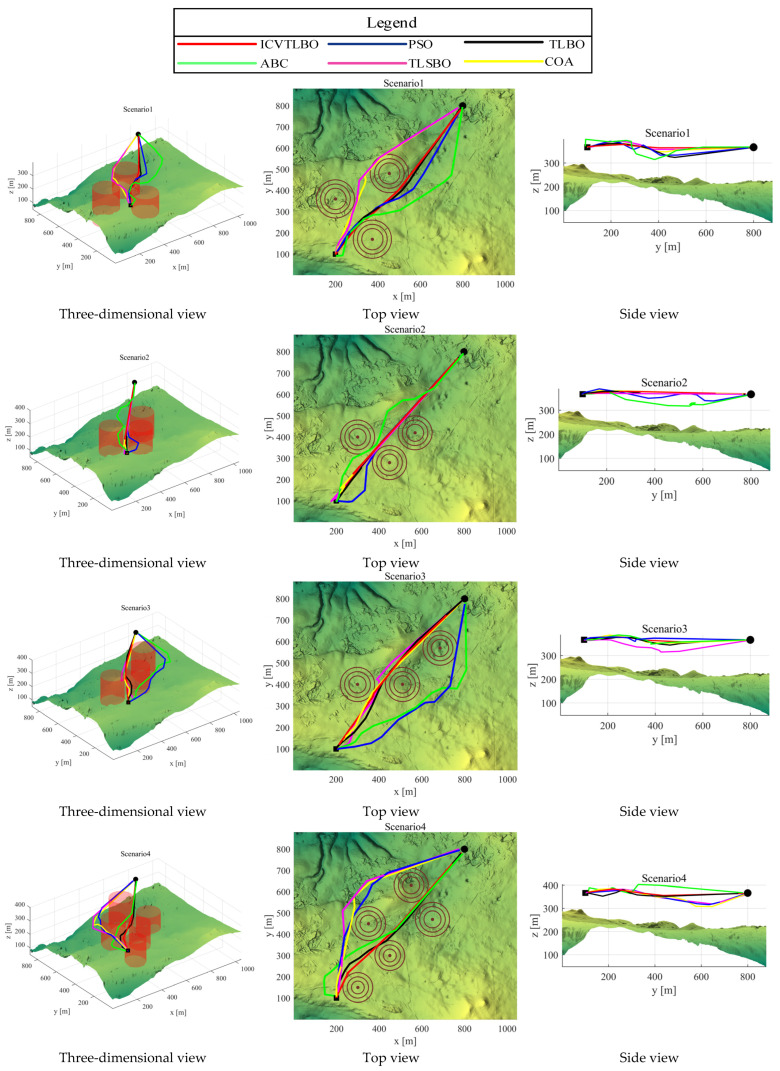

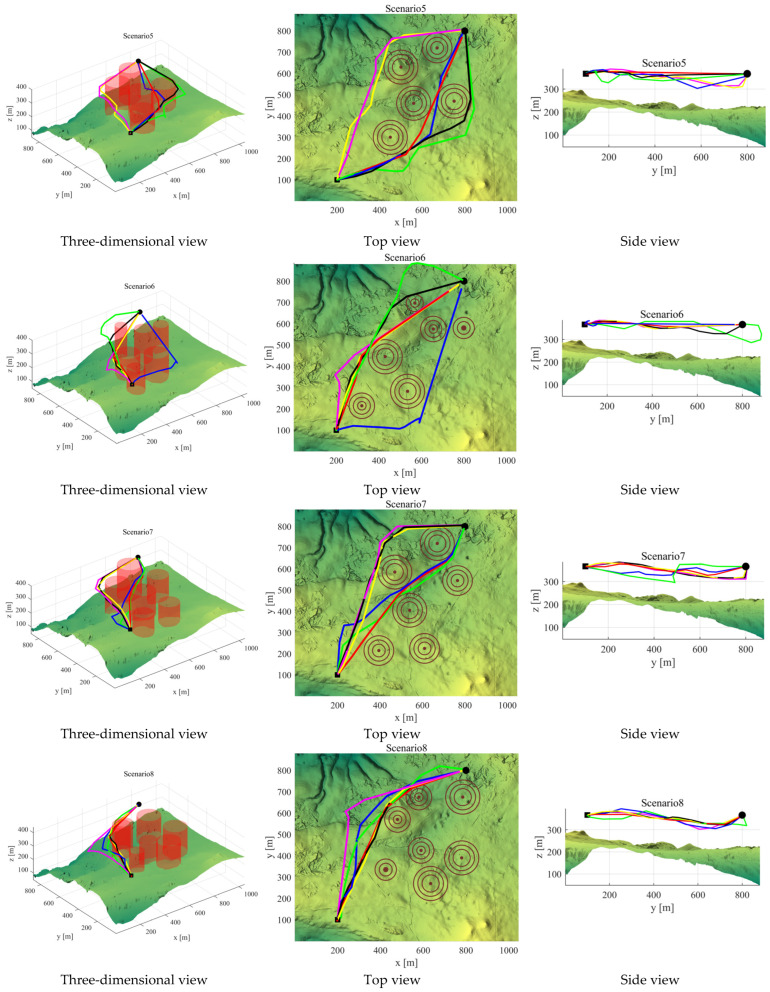

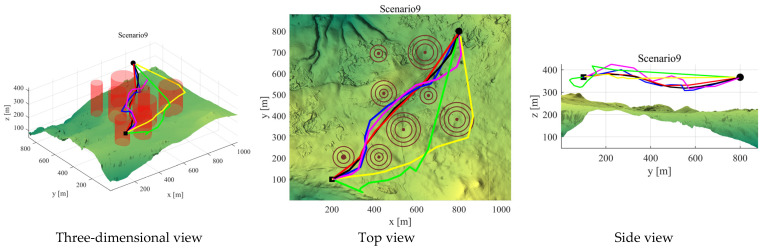
The corresponding three views.

In the current investigation, an exhaustive comparative analysis delves into the performance of various path-planning algorithms across nine distinct scenarios to elucidate the variances in solution accuracy and convergence rate among these algorithms. The empirical findings reveal that the ICVTLBO algorithm demonstrates superior performance metrics when benchmarked against other contending algorithms. Specifically, Figure 4 illustrates the three-dimensional, top, and side views of routes generated by different algorithms in nine path planning scenarios. While all tested algorithms can produce effective obstacle-free paths, the trajectories derived from ICVTLBO not only exhibit shorter lengths but also surpass in terms of smoothness, culminating in the highest path quality. In contrast, the pathways orchestrated by TLBO, TLSBO, PSO, ABC, and COA exhibit greater tortuosity, unequivocally attesting to the pronounced superiority of ICVTLBO in path optimization. Particularly commendable is ICVTLBO’s performance in intricate terrains, where the algorithm adeptly aids UAVs in maintaining a safe distance from obstacles while simultaneously devising short and secure navigation routes. This capability holds significant relevance for autonomous UAV flight within complex environments, as it not only ensures flight safety but also amplifies operational efficiency.

**Figure 5:**
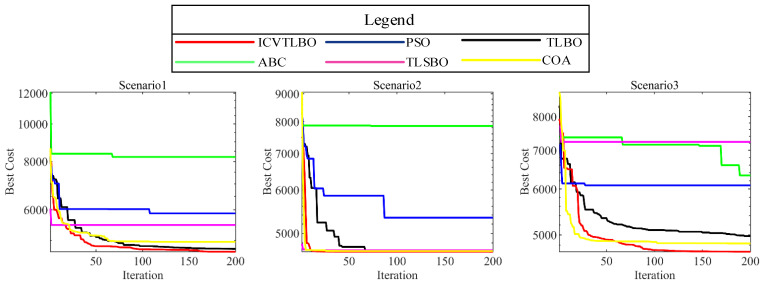

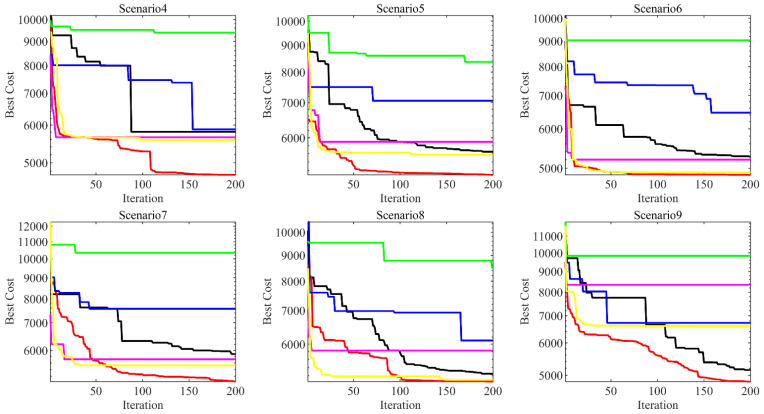
The corresponding convergence curves.

In the convergence curve diagrams across nine distinct scenarios, the ICVTLBO algorithm remarkably surpasses the other competitive algorithms in addressing the three-dimensional path planning issues for drones. A meticulous examination of the convergence behaviors of various algorithms under diverse conditions yields a clear conclusion that ICVTLBO significantly outperforms its predecessor, the conventional TLBO algorithm, in terms of convergence rate and global search capability. This fact robustly validates that the improved operator proposed in this study effectively remedies the deficiencies inherent to the TLBO algorithm. As depicted in Figure 5, aside from scenarios 4, 7, and 9, ICVTLBO rapidly converges to the optimal path within approximately 100 iterations in the remaining six scenarios. Among the six meta-heuristic algorithms compared, ICVTLBO exhibits the most rapid convergence speed. Further analysis reveals that as environmental complexity escalates, algorithms such as TLBO, TLSBO, PSO, and ABC are increasingly prone to becoming trapped in local optima, whereas ICVTLBO consistently approaches the global optimum unperturbed by the complexity of the path scenarios. This phenomenon strongly suggests that ICVTLBO possesses the most exceptional global exploration ability among the multitude of meta-heuristic algorithms. Synthesizing the analysis, ICVTLBO leads conspicuously ahead of the other comparative algorithms in convergence velocity, solution accuracy, and competence in navigating complex environments. Hence, ICVTLBO undoubtedly qualifies as a path planning algorithm replete with potential, meriting further research and widespread application.

### 5.3. Time Complexity Analysis

**Figure 6:**
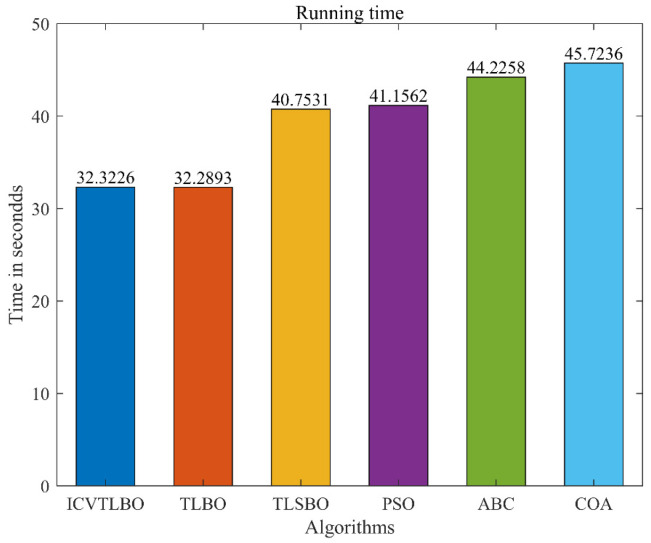
The runtime of the comparison algorithms across nine scenarios.

This section uses average execution time as the evaluation metric to verify the execution efficiency of ICVTLBO and the comparison algorithms, recording the total runtime of six comparison algorithms across nine scenarios. To ensure the reliability of the experimental data, each algorithm is independently run thirty times, and the average runtime is taken as the evaluation metric. The experimental results are shown in Figure 6.

As shown in Figure 6, when UAV path planning is performed in different scenarios, the average execution time of ICVTLBO is comparable to TLBO, while the time costs of TLSBO, PSO, ABC, and COA progressively increase. The average runtime of ICVTLBO exceeds TLBO by only 0.0333 s, and the time cost difference can be considered negligible, primarily due to the introduction of targeted interaction strategies in ICVTLBO during two stages. Although these strategies add some time cost, they significantly enhance convergence performance. Notably, ICVTLBO maintains efficiency like TLBO while demonstrating significant advantages. Through targeted interaction strategies, ICVTLBO more effectively explores the solution space, avoiding local optima and identifying better path planning solutions. Experimental results show that ICVTLBO converges quickly to high-quality solutions in various complex scenarios, significantly improving path planning efficiency. The paths planned by ICVTLBO are not only smooth and continuous but also better meet UAV flight performance constraints, enhancing flight safety and mission completion. In conclusion, ICVTLBO is comparable to TLBO in terms of time cost but shows significant advantages in global optimization, convergence speed, environmental adaptability, and path quality, providing an efficient and reliable solution to UAV path planning problems.

## 6. Conclusions

To address the challenging issue of three-dimensional drone path planning, this study proposes an innovative optimization algorithm, ICVTLBO, engineered to surpass the limitations of the original TLBO algorithm in terms of rapid convergence and avoidance of local optima. To augment the efficacy of the algorithm, ICVTLBO incorporates a diversified interactive strategy that not only bolsters its global exploration capabilities but also enhances population diversity, thereby circumventing the problem of premature convergence. Initially, by integrating a refined interactive strategy during the teacher phase within the iterative process, the ICVTLBO algorithm achieves a smooth transition from global exploration to local exploitation, significantly improving its search efficiency. Furthermore, the learner phase’s interactive strategy facilitates knowledge exchange among elite and average students within the population, hastening the algorithm’s convergence rate and enabling it to approach or attain the optimal solution in a shorter timeframe. Exhibiting its exceptional performance in the CEC2022 benchmark suite, the ICVTLBO algorithm ranked first across multiple metrics and significantly outperformed other comparative algorithms by minimizing mean values when solving functions. When applied to the 3D drone path planning problem, the algorithm consistently achieved the best fitness function values across nine diverse terrain scenarios, confirming its robust resilience and adaptability in complex environments. Additionally, the convergence curves of these algorithms indicate a significant advantage of ICVTLBO. Consistent experimental outcomes indicate that the ICVTLBO algorithm can secure highly competitive solutions under varying test environments, effectively resolving the 3D path planning problem for drones in intricate settings.

While the ICVTLBO algorithm demonstrates promising performance in current simulations, future research could extend from two key directions: (1) Cross-domain adaptive enhancement: Integrate deep reinforcement learning paradigms to strengthen dynamic decision-making under real-time environmental perturbations and explore federated learning frameworks for multi-UAV collaborative path planning in large-scale IoT deployments. (2) Resource-intelligence coupling: Develop energy-time-risk tri-objective optimization models that holistically consider 6G edge computing resource scheduling, UAV battery dynamics, and link stability thresholds, especially for time-sensitive sensing applications in smart cities.

## Figures and Tables

**Table 1:** Experimental results of each algorithm in CEC2022-20 dimensionality.

ID		ICVTLBO	ABC	PSO	TLBO	IDMO	COA	TLSBO
CEC2022-F1	Ave	5.9844E+02	1.0531E+05	1.4037E+03	1.0827E+04	9.4074E+02	4.7795E+04	2.2694E+09
	Std	2.2304E+02	3.1409E+04	1.1724E+03	5.2927E+03	5.8827E+02	1.5280E+04	8.3131E+09
CEC2022-F2	Ave	4.5100E+02	4.4401E+02	4.4198E+02	4.7231E+02	4.8429E+02	3.2083E+03	9.4564E+03
	Std	1.4510E+01	2.0319E+01	2.8288E+01	2.4697E+01	2.8425E+01	7.2381E+02	2.6647E+03
CEC2022-F3	Ave	6.0563E+02	6.0858E+02	6.4245E+02	6.0781E+02	6.4680E+02	6.8033E+02	7.3295E+02
	Std	5.1330E+00	1.9708E+00	1.1388E+01	5.5632E+00	1.1599E+01	1.1068E+01	1.8713E+01
CEC2022-F4	Ave	8.4886E+02	9.3649E+02	8.6816E+02	8.7224E+02	8.8000E+02	9.7697E+02	1.1276E+03
	Std	1.4606E+01	1.1319E+01	1.8654E+01	2.5415E+01	1.3429E+01	1.4443E+01	2.6200E+01
CEC2022-F5	Ave	1.1742E+03	1.7022E+03	2.3630E+03	1.1945E+03	2.2517E+03	3.4134E+03	1.4895E+04
	Std	2.2395E+02	3.1697E+02	6.0089E+02	2.1108E+02	2.2046E+02	4.5399E+02	2.9155E+03
CEC2022-F6	Ave	4.2836E+03	5.7301E+07	6.4952E+03	5.1311E+03	1.9120E+03	2.3640E+09	1.0595E+10
	Std	3.4020E+03	2.7501E+07	6.5458E+03	3.3756E+03	4.0189E+01	1.1287E+09	3.6338E+09
CEC2022-F7	Ave	2.0779E+03	2.1722E+03	2.1485E+03	2.0858E+03	2.1224E+03	2.2239E+03	2.6379E+03
	Std	4.4511E+01	2.2666E+01	6.0515E+01	2.7856E+01	2.9697E+01	5.3395E+01	1.4166E+02
CEC2022-F8	Ave	2.2337E+03	2.3166E+03	2.3556E+03	2.2436E+03	2.2556E+03	2.4816E+03	4.0736E+04
	Std	2.9026E+01	3.6915E+01	1.2007E+02	2.9410E+01	4.8165E+01	1.3656E+02	9.2986E+04
CEC2022-F9	Ave	2.4808E+03	2.5509E+03	2.4656E+03	2.4808E+03	2.4835E+03	3.3643E+03	4.3466E+03
	Std	3.3175E−05	3.1777E+01	8.5529E−02	2.9648E−02	3.1384E+00	2.9704E+02	7.3588E+02
CEC2022-F10	Ave	3.4209E+03	6.2997E+03	4.0802E+03	2.9326E+03	3.5941E+03	6.4448E+03	8.4497E+03
	Std	6.6812E+02	1.1971E+03	9.9318E+02	9.4021E+02	1.0239E+03	1.2701E+03	4.3588E+02
CEC2022-F11	Ave	2.8938E+03	3.0299E+03	2.9192E+03	2.9716E+03	3.2135E+03	8.5306E+03	1.5691E+04
	Std	1.0844E+02	4.8598E+01	1.5695E+02	1.2275E+02	2.2026E+02	6.3341E+02	3.3102E+03
CEC2022-F12	Ave	2.9638E+03	2.9000E+03	3.1618E+03	2.9737E+03	2.9812E+03	3.6408E+03	4.4677E+03
	Std	2.0809E+01	4.2210E−05	1.9490E+02	2.9156E+01	4.3255E+01	2.4318E+02	3.6014E+02
(W|T|L)		(8/4/0)	(1/11/0)	(1/11/0)	(1/11/0)	(1/11/0)	(0/12/0)	(0/0/12)
Mean		1.5417	4.0000	3.3333	2.6250	3.5833	5.9167	7.0000
Ranking		1	5	3	2	4	6	7

**Table 2:** Information about the location of obstacles.

Scenario Number	Obstacle Coordinates	Obstacle Radius
1	(374,170,120)	90
	(200,360,130)	90
	(454,480,130)	90
2	(300,400,150)	80
	(450,280,150)	80
	(570,420,150)	80
3	(300,400,150)	80
	(510,400,150)	80
	(685,570,150)	80
4	(300,150,150)	70
	(350,450,150)	80
	(550,630,150)	80
	(450,300,150)	70
	(650,470,80)	80
5	(450,300,120)	80
	(500,630,150)	80
	(560,460,150)	80
	(750,470,120)	80
	(670,720,150)	70
6	(320,215,150)	60
	(430,445,110)	80
	(535,283,150)	80
	(569,697,150)	40
	(656,575,150)	60
	(799,580,130)	50
7	(395,215,120)	70
	(470,585,150)	80
	(540,405,150)	80
	(610,225,120)	70
	(765,545,120)	70
	(670,720,150)	80
8	(425,335,120)	50
	(480,570,190)	60
	(590,425,100)	60
	(635,270,150)	80
	(780,390,150)	80
	(580,675,120)	60
	(785,675,100)	80
9	(255,205,170)	50
	(422,205,160)	60
	(445,506,170)	60
	(538,335,150)	80
	(656,496,120)	40
	(640,700,120)	80
	(420,695,150)	40
	(790,383,150)	80

**Table 3:** Cost function values for each algorithm.

Scenario	Performance	ICVTLBO	TLBO	TLSBO	PSO	ABC	COA
1	Mean	4755.4932	4761.1759	5377.9353	7345.7002	5663.2169	4838.9331
	Std	85.2959	43.1314	243.5628	622.5617	257.7655	71.0720
2	Mean	4627.4116	4645.3865	4655.3958	7110.8947	5520.6911	4646.9727
	Std	0.8281	12.1882	2.3520	401.2664	390.9906	5.9145
3	Mean	4694.1789	4852.1990	5510.8827	6597.3015	5987.5633	4702.6243
	Std	28.0143	83.4934	607.6555	257.9046	280.2343	14.2793
4	Mean	4948.8763	6356.4307	5765.7609	8952.2731	6667.5380	5261.9009
	Std	258.9795	425.8316	160.1771	746.9844	364.5507	343.6128
5	Mean	5160.3514	5640.0740	6159.2738	8685.0597	6387.2311	5299.7982
	Std	249.0467	76.7230	231.2509	594.0506	400.5888	207.7498
6	Mean	4888.1636	5330.3972	5114.2682	8744.5162	6688.7290	4889.7029
	Std	82.8671	105.0322	58.8425	490.0598	428.8033	14.1914
7	Mean	5413.4242	5730.6583	6395.8662	9284.9954	7186.9670	5462.2110
	Std	419.5398	187.7761	253.0665	818.3952	437.7184	262.3433
8	Mean	5131.2233	5183.2340	5682.2416	8580.1740	6512.4739	5162.1765
	Std	116.8275	45.7740	191.7747	545.7327	282.4209	84.7009
9	Mean	5209.7683	5598.9878	8426.2256	9275.8029	6715.5125	5237.2642
	Std	182.7870	294.7013	1220.5236	778.6092	434.9159	290.0866
(W|T|L)		(9/0/0)	(0/9/0)	(0/9/0)	(0/0/9)	(0/9/0)	(0/9/0)
Mean		1.00	3.00	3.89	6.00	4.89	2.22
Ranking		1	3	4	6	5	2

## Data Availability

The data that support the findings of this study are available from the corresponding author.
